# Letter to the Editor: Reply to Topkan et al

**DOI:** 10.1016/j.ctro.2025.100938

**Published:** 2025-02-25

**Authors:** Claudia Schweizer, Vratislav Strnad

**Affiliations:** a Department of Radiation Oncology, Universitätsklinikum Erlangen, Friedrich-Alexander-Universität Erlangen-Nürnberg (FAU), Erlangen, Germany; b Comprehensive Cancer Center Erlangen-EMN (CCC ER-EMN), Erlangen, Germany

**Keywords:** Early-stage oral cavity cancer, Postoperative radiotherapy, Interventional radiotherapy, Long-term toxicity after brachytherapy

## Abstract

We thank the colleagues Topkan and the co-authors for their valuable comments on our study. As they stated correctly, there for sure are more factors influencing the development of necrosis – nicotine and alcohol might also play an important role, for example. Also some hints point at the distance of the catheters being associated with risk of necrosis. Due to the fact that the risk factors influence each other in their effect on the risk of necrosis and usually have an additive effect and due to the generally retrospective data collections in published articles on interventional radiotherapy in the oral cavity, some risk factors for late side effects cannot be perfectly recorded and evaluated. In our understanding, not only the distance to the mandible, but also the bone volume which is affected by radiation dose must be considered. No specific dose constraints exist for the mandible when applying interventional radiotherapy. We are currently analyzing further dose parameters available within CT-based planning workflows and hope for more detailed information on how we can improve the implants. Nevertheless, prospective data is needed to sufficiently address toxicity issues in a larger cohort of patients with long-term follow-up. As far as the disease-free survival is concerned, we indeed estimated this according to the current practice in several other published data without taking the event of death into account. This is obvious when looking at our results. Still, we agree that the different ways of presenting freedom of recurrence throughout the literature makes comparison rather difficult and should be unified. We thank you for your remark and will consider this in our future work.

Dear Editor,

We thank the colleagues Topkan and the co-authors for their valuable comments on our study. As they stated correctly, there for sure are more factors influencing the development of necrosis – nicotine and alcohol might also play an important role, for example. Also some hints point at the distance of the catheters being associated with risk of necrosis [1]. Due to the fact that the risk factors influence each other in their effect on the risk of necrosis and usually have an additive effect and due to the generally retrospective data collections in published articles on interventional radiotherapy in the oral cavity, some risk factors for late side effects cannot be perfectly recorded and evaluated. In our understanding, not only the distance to the mandible, but also the bone volume which is affected by radiation dose must be considered. No specific dose constraints exist for the mandible when applying interventional radiotherapy. We are currently analyzing further dose parameters available within CT-based planning workflows and hope for more detailed information on how we can improve the implants. Nevertheless, prospective data is needed to sufficiently address toxicity issues in a larger cohort of patients with long-term follow-up. As far as the disease-free survival is concerned, we indeed estimated this according to the current practice in several other published data without taking the event of death into account [2], [3]. This is obvious when looking at our results. Still, we agree that the different ways of presenting freedom of recurrence throughout the literature makes comparison rather difficult and should be unified. We thank you for your remark and will consider this in our future work.

Kind regards,

Claudia Schweizer

Vratislav Strnad

## Declaration of Competing Interest

The authors declare that they have no known competing financial interests or personal relationships that could have appeared to influence the work reported in this paper.
